# Establishment of Quality Evaluation Method for Yinqiao Powder: A Herbal Formula against COVID-19 in China

**DOI:** 10.1155/2022/1748324

**Published:** 2022-11-25

**Authors:** Huimin Zhang, Lin Xu, Jian Song, Aijun Zhang, Xiao Zhang, Qingjun Li, Xinyan Qu, Ping Wang

**Affiliations:** ^1^Shandong Academy of Chinese Medicine, Jinan 250014, China; ^2^Shandong University of Traditional Chinese Medicine, Jinan 250355, China; ^3^Traditional Chinese Medicine Research Institute, Shandong Hongjitang Pharmaceutical Group Co Ltd., Jinan 250103, China; ^4^Laboratory of Immunology for Environment and Health, Shandong Analysis and Test Center, Qilu University of Technology (Shandong Academy of Sciences), Jinan 250014, China

## Abstract

Yinqiao powder, with significant anti‐inflammatory and antiviral effects, is a classical formula for the treatment of febrile diseases in China. During the SARS period in 2003, Yinqiao powder showed a good antipyretic effect. It also plays a major role in the treatment for COVID-19 in China. Although there are many studies on the chemical compositions and pharmacological effects of Yinqiao powder, there are few studies on the quality standard system of it. In our study, a systematic quality evaluation method of Yinqiao powder combining HPLC fingerprint with quantitative analysis of multi-components by single marker (QAMS) based on network pharmacology and UPLC-Q-Exactive-Orbitrap-MS was established for the first time. In the UPLC-Q-Exactive-Orbitrap-MS experiment, a total of 53 compounds were identified in the extract solution of Yinqiao powder. In addition, 33 blood components were characterized, 23 of which were prototypes. The results of network pharmacology analysis showed that Yinqiao powder may inhibit inflammatory responses by suppressing IL-6, CXCL2, TNF*α*, NF-*κ*B, etc., in the treatment of COVID-19. The HPLC fingerprint analysis of Yinqiao powder was conducted at 237 nm and 29 characteristic peaks were matched, 11 of which were identified. Forsythoside A was selected as the internal standard reference and double-wavelength (237 nm and 327 nm) was established in QAMS experiment. The repeatability was well under different conditions, and the results measured by QAMS were consisted with that of the external standard method (ESM), indicating that the QAMS method was reliable and accurate. The quality evaluation method of Yinqiao powder would be helpful to evaluate the intrinsic quality of Yinqiao powder more comprehensively, which is conducive to improve the quality standard of Yinqiao powder and provide a beneficial guarantee for the clinical treatment of COVID-19.

## 1. Introduction

Yinqiao powder is a well-known traditional Chinese medicine (TCM) formula in China. With significant anti-inflammatory and antiviral effects, it is clinically used for the treatment of influenza, infantile pneumonia, hand-foot-mouth disease, etc. [1–3]. In particular, during the SARS period in 2003, Yinqiao powder showed good antipyretic effect. Nowadays, coronavirus disease 2019 (COVID-19) is spreading around the world and causing severe respiratory illnesses and even death. Yinqiao powder is one of the heat-clearing and detoxification prescriptions recommended by the traditional Chinese medicine prevention and treatment plan for COVID-19 in Shaanxi, Jiangsu, Guangdong and Hubei provinces in China [4, 5]. However, the quality standard of Yinqiao powder is not perfect at present, so it is necessary to improve the quality control standard of Yinqiao powder.

Yinqiao powder is composed of ten herbal slices with complex ingredients [6]. The theory of serum pharmacochemistry of TCM believes that only components absorbed into bloodstream are likely to be virtually effective components [7]. Therefore, only by analyzing the serum components after oral administration and determining the direct acting components in the body of Yinqiao powder can fundamentally control the quality of the Yinqiao powder. The chemical components in TCM and biological samples could be accurately and quickly identified by UPLC-Q-Exactive-Orbitrap-MS technology, which provides a new method for improving the quality standard of Yinqiao powder [8]. So, UPLC-Q-Exactive-Orbitrap-MS was employed to detect the blood components of Yinqiao powder. In addition, HPLC fingerprint and quantitative analysis of multi-components by single marker (QAMS) are internationally recognized methods. The comprehensive information of Yinqiao powder could be obtained by HPLC fingerprints [9, 10] and QAMS can simultaneously determine the content of various components in Yinqiao powder through one reference substance [11–13].

**Figure 1:**
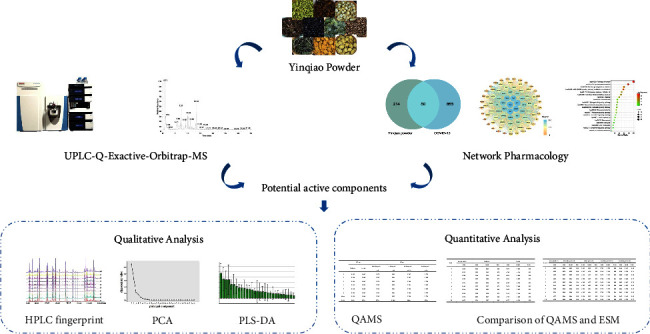
Flowchart of the established analytical strategy.

Yinqiao powder is a complex system with multi-components, multi-targets, and multi-action mechanisms through the joint action of various chemical components. The quality evaluation of it can not only use a single index. In this study, a qualitative and quantitative quality standard evaluation method of Yinqiao powder combining HPLC fingerprint with QAMS was established based on network pharmacology and UPLC-Q-Exactive-Orbitrap-MS technology. Firstly, UPLC-Q-Exactive-Orbitrap-MS was employed to detect the components in Yinqiao powder extract and rat blood. Meanwhile, network pharmacology was used to predict the possible pathways and components of Yinqiao powder in treating for COVID-19. Then, the common peaks of ten batches of Yinqiao powder were identified with the existing reference materials and the source of the common peaks was assigned through the establishment of HPLC fingerprint. Finally, the content of the potential active components screened in the first step was determined by the QAMS method with forsythoside A as the internal standard. The flowchart of the established analytical strategy is shown in Figure 1.

## 2. Materials and Methods

### 2.1. Chemicals and Reagents

Caffeic acid, hesperidin, rutin, liquiritin, cynaroside, and forsythoside A were provided by National Institutes for Food and Drug Control (Beijing, China). Arctiin, neochlorogenic acid, chlorogenic acid, isochlorogenic acid A, and isochlorogenic acid C were purchased from Shanghai Yuanye Bio-Technology Co., Ltd. (Shanghai, China). As for phillyrin, it was provided by Chengdu Chroma-Biotechnology Co., Ltd. (Chengdu, Sichuan Province, China). The detailed information of the above mentioned standard materials is listed in Table S1. All the herbal slices of Yinqiao powder were purchased from Beijing Tongrentang drugstore (Beijing, China), and information of these herbal slices is shown in Table 1.

The deionized water was obtained from Watsons (Watsons Food and Beverage Co., Ltd., Guangzhou, China). Acetonitrile (HPLC grade), formic acid (HPLC grade), and methanol (HPLC grade) used in UPLC analysis were provided by Fisher Scientific (Thermo Fisher, CA, USA). In quantitative analysis, acetonitrile (HPLC grade) and methanol (HPLC grade) were purchased from BCL International Trading Co., Ltd. (USA), and methanol (AR) was obtained from Tianjin Fuyu Fine Chemical Co., Ltd. (Tianjin, China).

### 2.2. Network Pharmacology Analysis

#### 2.2.1. Prediction of Chemical Components and Related Targets of Yinqiao Powder

The chemical components and related targets of Yinqiao powder were predicted using TCMSP (https://tcmspw.com/tcmsp.php) and Swiss Target Prediction platform (https://www.swisstargetprediction.ch). Oral bioavailability (OB) ≥30% and drug-likeness (DL) ≥0.18 were used as the screening criteria. The active ingredients of Yinqiao powder that are not included in TCMSP or do not meet the screening standards were added according to the Pharmacopoeia of the People's Republic of China (Ch. P) and literature. The herbal slices-chemical composition-gene data sheet of Yinqiao powder was established based on above information.

#### 2.2.2. Prediction of Related Targets of COVID-19

The targets related to COVID-19 were obtained by searching in DisGeNET (https://www.disgenet.org/), DrugBank (https://go.drugbank.com/), GeneCards (https://www.genecards.org/), and OMIM (https://www.omim.org/) databases and screened with the keywords of “COVID-19,” “Coronavirus disease 2019,” “novel coronavirus pneumonia,” “coronavirus,” “SARS-coronavirus,” “Severe Acute Respiratory Syndrome” and “Coronavirus Infections.” In GeneCards database, score ≥10 was used as the screening criteria.

Then, the intersection targets of Yinqiao powder and COVID-19 were obtained using the online platform of Bioinformatics (https://www.bioinformatics.com.cn/) through Venn diagram analysis.

#### 2.2.3. Construction and Analysis of the PPI Network

The intersection targets of Yinqiao powder and COVID-19 were submitted to STRING database (https://cn.string-db.org/, Version 11.5) for the construction and analysis of the protein-protein interaction (PPI) network. The organism was restricted to *Homo sapiens* and the minimum required interaction score was set as medium confidence (0.400). Then, the result of *TSV* format was exported and imported into Cytoscape 3.8.2 software for further analysis.

#### 2.2.4. GO and KEGG Enrichment Analysis

The potential genes of Yinqiao powder for the treatment of COVID-19 were imported into Metascape database (https://metascape.org) for Gene Ontology (GO) and Kyoto Encyclopedia of Genes and Genomes (KEGG) enrichment analysis. Species of *Homo sapiens* and *p* < 0.01 were set as the filter criteria. The visual analysis of GO and KEGG enrichment results was performed using Bioinformatics online platform (https://www.bioinformatics.com.cn/).

### 2.3. Instruments and Conditions

#### 2.3.1. Instruments

Ultimate 3000 ultra-high performance liquid chromatography system (Thermo Fisher, CA, USA) was connected to a Q-Exactive Orbitrap tandem mass spectrometer (Thermo Fisher, CA, USA) via an ESI source. Oil-Free Air Compressors (AC-1Y) was purchased from Beijing Purkinje General Instrument Co., Ltd. (Beijing, China). High-speed refrigerated centrifuge was acquired from Heraeus Holding GmbH (GER). MX-F vortex Mixers was provided by Wuhan Servicebio Technology Co., Ltd. (Wuhan, China). Both CNC ultrasonic cleaner (Q-250D, Kun Shan Ultrasonic Instruments Co., Ltd.) and 1/10000 electronic scale (XS205DU, Mettler Toledo International Trading (Shanghai) Co., Ltd.) were also used in quantitative analysis.

#### 2.3.2. Conditions

The chemical identification analysis was performed on UPLC-Q-Exactive-Orbitrap-MS. Samples were firstly separated on HALO-C18 Column (2.1 mm × 100 mm, 2.7 *μ*m, AMT Company). The column temperature was set at 30°C. The flow rate was 0.3 mL·min^−1^ and the sample injection volume was 5 *μ*L. The mobile phase consisted of 0.1% formic acid-water (*A*) and acetonitrile (*B*). The optimized gradient elution was as follows: 0–2 min, 2% *B*; 2–32 min, 2%–75% *B*; 32–33 min, 75%–2% *B*; 33–35 min, 2% *B*.

The mass spectrometer was operated in both positive and negative ion modes. The spray voltage was maintained at 3.5 kV in the positive ion mode and 3 kV in the negative ion mode. Full MS spectra were acquired with resolution at 70,000 and AGC target at 1*e*^6^. MS/MS fragments (spectra) were performed with resolution at 17,500 and AGC target at 1*e*^5^. The stepped normalized collision energy (NCE) was set at 20, 40, and 60. The following parameter settings were used: capillary temperature of 320°C, heater temperature of 300°C, sheath gas velocity of 35 arb, auxiliary gas flow rate of 10 arb, and mass range of *m/z* 80–1200.

In the fingerprint analysis and QAMS experiments, chromatographic separation was performed on a Thermo Syncronis Column (4.6 mm × 250 mm, 5 *μ*m, Thermo Fisher, CA, USA). Water with 0.1% phosphoric acid (*A*) and acetonitrile (*B*) were used as mobile phase. The flowing gradient was applied at 30°C and set as follows: 0–5 min, 7%-8% *B*; 5–15 min, 8%–10% *B*; 15–20 min, 10%–11% *B*; 20–35 min, 11%–16% *B*; 35–70 min, 16%–18% *B*; 70–90 min, 18%–20% *B*; 90–100 min, 20%–23% *B*; 100–130 min, 23%–60% *B*. The flow rate was 1 mL·min^−1^ and the sample injection volume was 5 *μ*L. Under the above mentioned chromatographic conditions, peak area of each component at 237 nm was measured and recorded for fingerprint spectrum, while the peak area of each component at 237 nm and 327 nm was measured and recorded for QAMS. The relative correction factors (RCFs) of parallel experiments were recorded and calculated using two high-performance liquid chromatographs (Thermo UltiMate3000 (1), Thermo UltiMate3000 (2)) and two chromatographic columns (Hypersil ODS2 (250 mm × 4.6 mm, 5 *μ*m), Thermo Syncronis C_18_ (250 mm × 4.6 mm, 5 *μ*m)).

### 2.4. Animal Experiments

Healthy male and female Wistar rats (SPF grade, 240 ± 10 g) were purchased from Beijing Vital River Laboratory Animal Technology Co., Ltd. (License No.: SCXK (Jing) 2016-0006) and housed for 4 days of acclimation before the experiment. The animal research was conducted in accordance with international rules for animal experimentation and internationally recognized ethical principles for the use and care of laboratory animals. The animal study protocol was approved by the Ethics Committee of Shandong Academy of Chinese Medicine.

Serum sample preparation: twelve Wistar rats were divided into an experimental group and a blank group (half of the rats in each group were female). Eight hours before the administration, rats were fasted with free access to water. Rats in the experimental group were given 4 mL of extract of Yinqiao powder, while rats in the blank group were given the same volume of normal saline (0.9% NaCl). 0.5 mL blood samples were collected from the jugular vein of rats at 30 min, 60 min, 90 min, 120 min, and 180 min, respectively after administration. After standing for 1 h, each blood sample was centrifuged at 3,000 rpm for 15 min to obtain the serum. The serum samples were stored at −80°C until further pre-treatment.

The freeze-thaw of all serum samples was carried out at 4°C. Each serum sample (200 *μ*L) was mixed with acetonitrile (600 *μ*L), and then centrifuged at 13,000 rpm at 4°C for 15 min. Afterward, the supernatant was dried with a stream of nitrogen at room temperature to obtain the residue. Then, it was redissolved in 70% methanol‐water (100 *μ*L) and centrifuged at 15,000 rpm at 4°C for 15 min. The supernatant was used for further analysis.

### 2.5. Preparation of Sample Solutions and Negative Sample Solutions

#### 2.5.1. Sample for Animals

100 g of Yinqiao powder was prepared according to the Ch. P of the 2020 edition [6]. In a volumetric flask (2000 mL), Yinqiao powder was soaked in 10 times of water for 1 h and filtered after reflux extraction for 1 h. The filtrate was collected, and then the water with residues (1 : 8, w/v) was boiled for an additional 1 h. Two batches of filtrate were mixed. After collecting the volatile oil, the concentration of the filtrate was concentrated to 2 g·mL^−1^. Afterward, the filtrate cooled to room temperature was mixed with the volatile oil and the mixture was used in intragastric administration of rats.

#### 2.5.2. Sample for UPLC-Q-Exactive-Orbitrap-MS

Yinqiao powder (10 g) was extracted according to the above method. The filtrate was collected and then concentrated to 100 mL. The mixture of the abovementioned solution (1 mL) and methanol (1 mL) was processed by an ultrasonic assisted extraction (UAE) method for 15 min and then centrifuged at 15,000 rpm at 4°C for 15 min. The supernatant was isolated for further analysis.

#### 2.5.3. Sample for Fingerprint and QAMS Analysis

Sample of Yinqiao powder: 0.5 g of Yinqiao powder was accurately weighed and placed in a volumetric flask (25 mL). The volumetric flask was filled with 70% methanol-water. After being processed by the UAE method for 30 min, the solution of Yinqiao powder was cooled to room temperature and then filtered to obtain the filtrate. Afterward, the filtrate was filtered through a 0.45 *μ*m millipore filter membrane (organic-system) for further analysis.

Preparation of negative control samples: the negative control samples of *Lonicerae Japonicae Flos*, or *Forsythiae Fructus*, or *Arctii Fructus*, or *Lonicerae Japonicae Flos* and *Arctii Fructus* were respectively produced as same as “Sample of Yinqiao powder” for further analysis.

### 2.6. Preparation of the Standard Solutions

Neochlorogenic acid, chlorogenic acid, forsythoside A, isochlorogenic acid A, isochlorogenic acid C, phillyrin, and arctiin were accurately weighed and dissolved in 50% methanol. The finally concentration of them was 7.7 *μ*g·mL^−1^, 96 *μ*g·mL^−1^, 296 *μ*g·mL^−1^, 65 *μ*g·mL^−1^, 15.2 *μ*g·mL^−1^, 27.5 *μ*g·mL^−1^, and 113 *μ*g·mL^−1^, respectively. All standard solutions were stored at 4°C.

### 2.7. Data Processing and Analysis

The UPLC-MS data were handled by Compound Discoverer 3.2 software, and compared with ChemSpider, Thermo's Chinese Medicine database and mzCloud database. The HPLC fingerprint analysis was performed using the “Similarity Evaluation System of Traditional Chinese Medicine Chromatographic Fingerprint” software (2004, edition). SPSS17.0 software was used for principal component analysis (PCA) of the key components affecting the quality of Yinqiao powder.

## 3. Results and Discussion

### 3.1. Method Development

#### 3.1.1. Optimization of Chromatographic Conditions for Fingerprint and QAMS

We examined different mobile phases according to related literatures [14, 15]. The results showed that the baseline was more stable when acetonitrile-0.1% phosphoric acid water was used as mobile phase compared with methanol-0.1% phosphoric acid water. Therefore, acetonitrile-0.1% phosphoric acid water was chosen as mobile phase. The elution gradient was based on the conditions in Section 2.3.2. Under this gradient, there were more chromatographic peaks of Yinqiao powder with better resolution and larger peak area.

Furthermore, in order to select detection wavelengths, the chromatograms at 237 nm, 279 nm, 327 nm, and 365 nm were compared. The results suggested that information collected at 237 nm was the most abundant, and peak area and resolution were better, which could well reflect the characteristics of Yinqiao powder in fingerprint analysis. Furthermore, in QAMS experiment, forsythoside A, phillyrin and arctiin had better asymmetry and resolution at 237 nm. The resolution of neochlorogenic acid, chlorogenic acid, forsythoside A, isochlorogenic acid *A*, and isochlorogenic acid *C* were greater than 1.5 without interference of impurity peaks at 327 nm. However, there was no absorption for phillyrin and arctiin at 327 nm. Therefore, the content detection was performed at 237 nm and 327 nm, while 237 nm was selected as the detection wavelength in the fingerprint analysis.

#### 3.1.2. Optimization of Experimental Conditions for Fingerprint Analysis

The Yinqiao powder was extracted by the ultrasonic extraction method according to Ch. P [6] and related literatures [16, 17]. In our study, 50% methanol, 80% methanol, and 100% methanol were investigated. The results showed that there was no significant difference on the number of components extracted by the three solvents and on the resolution of chromatographic peaks. However, the chromatographic peak area of the samples extracted with 50% methanol was largest under the same chromatographic conditions. Thus, 50% methanol was chosen as the extraction solvent.

In addition, the influence of solvent peaks on the results must be taken into account [18]. So, 50% methanol was injected according to the above chromatographic conditions. The solvent peaks of 0–5 min was sheared during peak matching based on the results of the chromatogram. Then, the chromatograms of 10 batches of Yinqiao powder were matched with common peaks. As a result, there were 112 common peaks under the unrestricted conditions. However, the peak area of most of the common peaks was small and the signal-to-noise ratio (SNR) did not meet the requirements. When the peak area was greater than 0.3, there were 38 common peaks and 11 chromatographic peaks could be identified. However, some peaks with small area had low resolution and asymmetry. For the further filter, the condition that peak area was greater than 0.4 was carried out. A total of 29 common peaks were obtained, 11 of which could be identified and the resolution and asymmetry of each common peak were good. In the end, peak area greater than 0.4 was determined as the screening condition.

#### 3.1.3. Optimization of Experimental Conditions for QAMS

Forsythoside A showed absorption at 237 nm and 327 nm, and the peak shape and resolution were good. Besides, it had high content and stable property in Yinqiao powder. Therefore, forsythoside A was selected as the internal standard reference.

For the location of chromatographic peaks for each component, relative retention value and retention time differences are the common qualitative parameters [19]. In our study, the two parameters of each component relative to the internal standard reference were calculated. Finally, neochlorogenic acid and chlorogenic acid were located by retention time differences while isochlorogenic acid A, isochlorogenic acid C, phillyrin, and arctiin were located by a relative retention value.

### 3.2. UPLC-Q-Exactive-Orbitrap-MS Technology

#### 3.2.1. Analysis of Reference Substances

The total ion chromatograms (TICs) in positive and negative ion modes of reference substances were analyzed. The results indicated that the information of each component responded better in negative ion mode. The detailed information of reference substances is listed in Table 2.

Neochlorogenic acid and chlorogenic acid are isomers of each other with the same theoretical [M − H]^−^ ion and fragment ions. Chlorogenic acid is a phenolic acid compound produced by the reaction of caffeic acid and quinic acid, and generated the [M − H]^−^ ion at *m/z* 353.0876. The fragment ions at *m/z* 191.0554 and *m/z* 179.0341 were detected in ESI-MS/MS spectrum, which were [M − H]^−^ ions of quinic acid and caffeic acid, respectively. Isochlorogenic acid A and isochlorogenic acid C, with the same [M − H]^−^ ion at *m/z* 515.1195, belong to the dicaffeoylquinic acid. The positions of their functional group were different. In their ESI-MS/MS spectrums, characteristic fragment ions were detected, including *m/z* 353.0882, *m/z* 191.0554, and *m/z* 179.0446. Arctiin and phillyrin are also isomers of each other. The [*M* + HCOO]^−^ ion at *m/z* 579.2091 was formed by the combination of arctiin with a formate according to published literatures [20, 21] and spectra.

#### 3.2.2. Analysis of Active Ingredients in Yinqiao Powder

According to the above analysis methods and related literatures, a total of 53 compounds were identified in the extract solution of Yinqiao powder, including 14 flavonoids, 8 organic acids, 8 amino acids, 7 phenylethanoid glycosides, 6 isoflavones and isoflavone glycosides, 4 iridoids, 2 lignans, 1 coumarin, and 3 other compounds (Table 3).

#### 3.2.3. Analysis of Blood Components in Yinqiao Powder

A total of 33 blood components were identified by comparing the chemical components in Yinqiao powder extract, blank serum, and drug serum, 23 components of which were found in both Yinqiao powder extract and drug serum and other 10 components were only present in the drug serum (Table 4). According to related literatures [38–41], chlorogenic acid, isochlorogenic acid A, isochlorogenic acid C, phillyrin, forsythoside A, arctiin, liquiritin, neochlorogenic acid, cynaroside, rutin, and hesperidin can be absorbed into blood in the form of prototype. It has been reported that phillyrin and forsythoside A have good antiviral and immune regulation effects [42–44], neochlorogenic acid, isochlorogenic acid A, and isochlorogenic acid C have antiviral and anti-inflammatory activities [45, 46], while cynaroside has the effect of inhibiting influenza virus [47]. Liquiritin, hesperidin, and rutin have obvious anti-inflammatory effects [48–50]. The content of the abovementioned components in the Yinqiao powder extract is relatively high, and all of them can be absorbed into the blood with potent anti-inflammatory and antiviral effects. Therefore, these components can be used as indicators to further improve the quality standard of Yinqiao powder.

### 3.3. Network Pharmacology Analysis of Yinqiao Powder

#### 3.3.1. Target Predication of Yinqiao Powder and COVID-19

**Figure 2:**
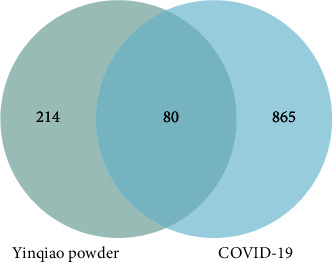
Venn diagram analysis of Yinqiao powder and COVID-19.

A total of 136 components and 294 related targets of Yinqiao powder were obtained according to Ch. P, related literature combined with the TCMSP database. At the same time, GeneCards, OMIM, DisGeNET, and DrugBank databases were used to screen the targets of COVID-19. As a result, 945 targets were finally retained after removing duplicates. Then, Venn diagram analysis was performed on the targets of the two and it was found that there were 80 intersection targets (Figure 2).

#### 3.3.2. Construction and Analysis of the PPI Network

**Figure 3:**
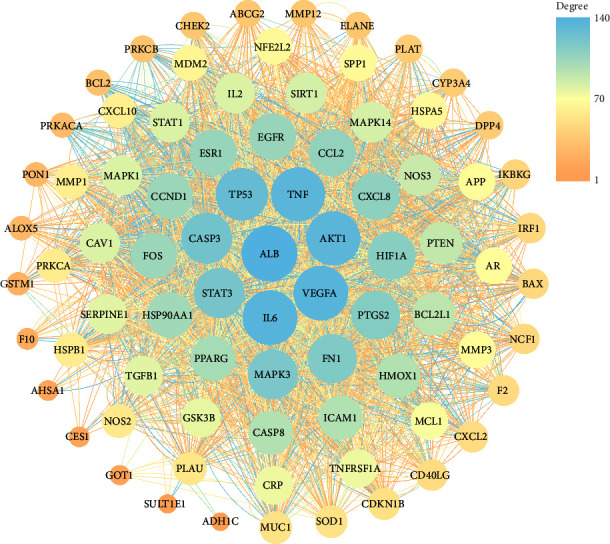
PPI network of intersection targets. The area of the node in Figure 3 is proportional to the degree value.

The 80 intersecting targets of Yinqiao powder and COVID-19 were submitted to STRING database to construct the PPI network. Then, the result was imported into Cytoscape 3.8.2 software for further analysis. In the end, a PPI network of Yinqiao powder intreating for COVID-19 with 80 nodes and 2824 edges was acquired, as shown in Figure 3. The area of the node in the figure is proportional to the degree value. So, it can be seen intuitively that ALB, IL6, VEGFA, AKT1, TNF, TP53, CASP3, and STAT3 may play important roles in the process of Yinqiao powder in treating for COVID-19.

The chemical components corresponding to the intersection targets were initially identified as the effective active components of Yinqiao powder. A total of 126 components were found in our study, including quercetin, luteolin, kaempferol, chlorogenic acid, isochlorogenic acid A, isochlorogenic acid C, arctiin, neochlorogenic acid, forsythoside A, liquiritin, and so on. Studies have shown that chlorogenic acid and phillyrin could inhibit the production of TNF-*α*, IL-1*β*, IL-6 and alleviate lung infection in mice [43, 51]. Neochlorogenic acid could reduce the production of TNF-*α*, IL-6, and NO, further inhibit the protein expression of iNOS, COX2, TNF-*α*, IL-6, and attenuate the inflammatory response by activating the AMPK/Nrf2 signaling pathway [52]. Arctiin, together with daidzein, glycyrrhizic acid and liquiritin, can inhibit pneumonia by enhancing necroptosis and partial autophagy associated with plc *γ*1 phosphorylation in natural killer cells [53]. Forsythoside A could ameliorate lipopolysaccharide-induced pathological damage, decreased serum levels of TNF-*α* and IL-6, and inhibited macrophage infiltration in the lungs of acute lung injury mice [54].

#### 3.3.3. GO and KEGG Enrichment Analysis

**Figure 4:**
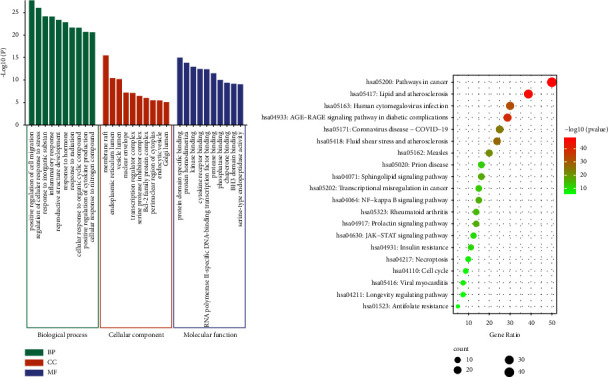
GO (a) and KEGG (b) enrichment analysis of Yinqiao powder in treating for COVID-19.

Furthermore, the 80 targets were submitted to Metascape for GO and KEGG enrichment analysis. *P* < 0.01 was used as the screening criterion. As a result, 1570 GO items were enriched, including 1413 in biological processes (BP), 55 in cellular components (CC), and 102 in molecular functions (MF). At the same time, 191 KEGG pathways were obtained. The visual analysis of the top 10 entries of GO and top 20 pathways of KEGG enrichment analysis is shown in Figure 4.

The GO enrichment analysis results showed that positive regulation of cell migration, regulation of cellular response to stress, response to inorganic substance, inflammatory response, and reproductive structure development were involved in BP. In the aspect of CC, it was mainly included membrane raft, endoplasmic reticulum lumen, vesicle lumen, nuclear envelope, transcription regulator complex, etc. The top 5 items of MF were protein domain specific binding, protein homodimerization activity, kinase binding, cytokine receptor binding, and RNA polymerase II-specific DNA-binding transcription factor binding.

The KEGG enrichment results suggested that the pathways of Yinqiao powder in the treatment for COVID-19 were mainly related to inflammation, such as IL-17 signaling pathway, TNF signaling pathway, PI3K-Akt signaling pathway, NF-kappa B signaling pathway, ErbB signaling pathway, coronavirus disease—COVID-19, and so on (Table S2). COVID-19 is caused by the severe acute respiratory syndrome coronavirus 2 (SARS-CoV 2). SARS-CoV 2 infects alveolar epithelial cells through the angiotensin-converting enzyme 2 (ACE2) receptor, resulting in increased serum levels of free angiotensin II (Ang II). The increased serum level of free Ang II promotes activation of the NF-kappa B pathway via Ang II type 1 receptor (AT1R), followed by interleukin-6 (IL-6) production. Studies have shown that the serum levels of IL-6, IL-17, and TNF-*α* in the COVID-19 patients were significantly higher than those in the control group [55, 56]. The damage associated with inflammatory autoimmune diseases can be reduced by inhibiting IL-17 [57]. Robinson et al. retrospectively explored the potential of anti-TNF in modulating COVID-19-related inflammation and concluded that anti-TNF therapies could reduce levels of pro-inflammatory cytokines associated with poor COVID-19 outcomes [58]. In addition, NF-*κ*B is involved in the regulation of immunity, inflammation and cell survival, which could be activated by TNF-*α*, IL-1, etc. Numerous studies have demonstrated the potential therapeutic effect of inhibiting the NF-*κ*B pathway in relieving severe forms of COVID-19 [59, 60]. The ErbB signaling pathway can regulate cell proliferation, migration, differentiation, apoptosis, and cell movement by mediating the PI3K-Akt pathway, JAK-STAT pathway, and MAPK signaling pathway. It was speculated that Yinqiao powder could inhibit JAK-STAT signaling through IL-6, which could reduce inflammatory responses and alleviate lung injury [61]. EGFR and EGBB2 are members of the epidermal growth factor receptor family. In the ErbB signaling pathway, they can directly or indirectly activate PI3K, thereby improving severe pneumonia by inhibiting the PI3K-Akt signaling pathway. Therefore, Yinqiao powder may inhibit the inflammatory response by inhibiting IL-6, CXCL2, MMP1, TNF*α*, NF-*κ*B, etc., in the treatment of COVID-19.

### 3.4. Qualitative Analysis by Fingerprint and Multiple Statistical Strategies

**Figure 5:**
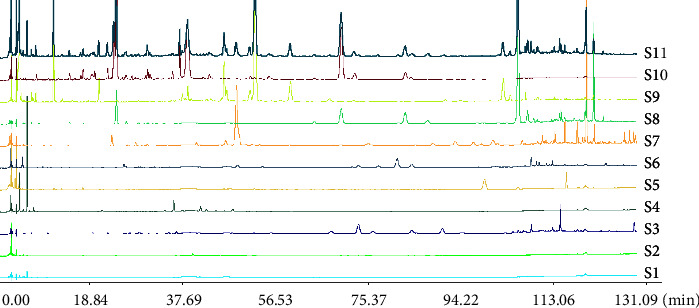
Matching graph of chromatogram between *yinqiao powder* and single drug. *S*1. *Phragmitis Rhizoma*, *S*2. *Platycodonis Radix*, *S*3. *Schizonepetae Spica*, *S*4. *Lophatheri Herba*, *S*5. *Sojae Semen Praeparatum*, *S*6. *Menthae Haplocalycis Herba*, *S*7. *Glycyrrhizae Radix et Rhizoma*, *S*8. *Arctii Fructus*, *S*9. *Forsythiae Fructus*, *S*10. *Lonicerae Japonicae Flos*, *S*11. Yinqiao powder.

Yinqiao powder and single drug samples were prepared according to the method given in Section 2.5.3, and standard solutions were prepared according to the method given in Section 2.6. The samples were detected under chromatographic conditions of fingerprint analysis in Section 2.3.2; then, the UPLC fingerprint analysis was performed using the “Similarity Evaluation System of Traditional Chinese Medicine Chromatographic Fingerprint” software (2004, edition). The matching results are shown in Figure 5.

#### 3.4.1. Precision, Stability, and Repeatability

The same batch of Yinqiao powder solution was injected 6 times for the validation of precision and the stability was validated by analyzing the sample of Yinqiao powder at 0, 3, 6, 9, 12, and 24 h at room temperature. It can be seen from the results that RSDs of the retention time of each common peak was 0.01%∼0.08%, and RSDs of relative peak area was 0.32%∼2.19%, indicating that the precision of the instrument was excellent and the sample was stable in 24 hours.

Meanwhile, six samples of the same batch of Yinqiao powder were accurately weighed, prepared, and tested in parallel to test the repeatability. The results showed that RSDs of each common peak were less than 0.15% and the relative peak area was less than 2.52%, which indicated that the repeatability was good.

#### 3.4.2. Similarity Evaluation of Fingerprint of Yinqiao Powder

Ten batches of Yinqiao powder sample were prepared and tested, and the chromatograms of each batch were recorded. The HPLC fingerprint analysis was performed using “Similarity Evaluation System of Traditional Chinese Medicine Chromatographic Fingerprint” software (2004, edition). *S*1 was chosen as the reference peak, the fingerprint of Yinqiao powder was established by median method. The results suggested that the similarity of 10 batches of Yinqiao powder was more than 0.95 (Table S3), indicating that the quality of Yinqiao powder was stable.

#### 3.4.3. Confirmation of Common Peaks in Fingerprint

**Figure 6:**
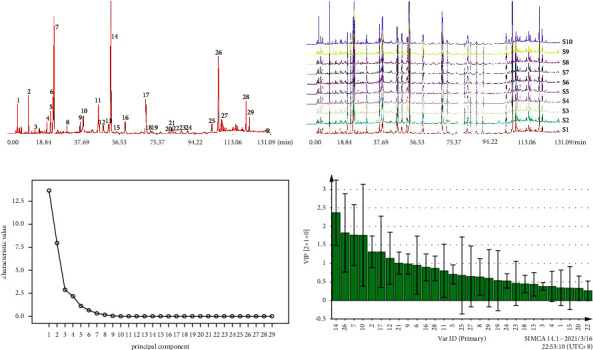
Reference spectrum of *Yinqiao powder* (a), HPLC fingerprint of 10 batches of yinqiao powder (b), scree plot of principal component of PCA (c), and VIP value of common peaks of PLS-DA (d).

29 common peaks were determined and 11 common peaks can be identified by comparing the chromatograms of 10 batches of Yinqiao powder. The 11 identified peaks were as follows: peak 3 was neochlorogenic acid, peak 7 was chlorogenic acid, peak 12 was glycyrrhizin, peak 13 was rutin, peak 14 was forsythoside A, peak 15 was cynaroside, peak 17 was isochlorogenic acid A, peak 19 was hesperidin, peak 21 was isochlorogenic acid C, peak 25 was phillyrin, and peak 26 was arctiin. The reference spectrum and fingerprint spectrum of 10 batches of Yinqiao powder are shown in Figures 6(a) and 6(b), the relative retention time is shown in Table S4, and the median retention time and average peak area are shown in Table S5.

#### 3.4.4. Attribution of Common Peaks in Fingerprint

The reference spectrum of Yinqiao powder was compared with samples of single medicinal materials and negative control medicinal materials. Peak 1 came from *Rhizoma Phragmitis* and *Lophatheri Herba*. Peak 7 (chlorogenic acid), peak 17 (isochlorogenic acid A), and peak 21 (isochlorogenic acid C) came from *Lonicerae Japonicae Flos* and *Arctii Fructus*. Peak 10 came from *Lonicerae Japonicae Flos* and *Forsythiae Fructus*. Peak 19 (hesperidin) was from *Menthae Haplocalycis Herba* and *Schizonepetae Spica*, and peak 22 was from *Lonicerae Japonicae Flos*, *Menthae Haplocalycis Herba* and *Schizonepetae Spica.* Peak 28 existed in 10 medicinal materials, and peak 29 came from *Forsythiae Fructus*, *Arctii Fructus* and *Glycyrrhizae Radix et Rhizoma*. Peak 12 (glycyrrhizin) was the exclusive peak of *Glycyrrhizae Radix et Rhizoma*, peak 24 was the exclusive peak of *Schizonepetae Spica*, peak 20 was the exclusive peak of *Menthae Haplocalycis Herba*. Additionally, peaks 3 (neochlorogenic acid), 5, 6, 8, 9, and 18 were the exclusive peaks of *Lonicerae Japonicae Flos*, and peaks 2, 4, 11, 13, 14 (forsythoside A), 16, 25 (phillyrin), and 27 were the specific peaks of *Forsythiae Fructus*. Peaks 23 and 26 (arctiin) were the characteristic peaks of *Arctii Fructus*. The results showed that most of the common peaks came from *Lonicerae Japonicae Flos*, *Forsythiae Fructus* and *Arctii Fructus*, indicating that *Lonicerae Japonicae Flos*, *Forsythiae Fructus*, and *Arctii Fructus* play important roles as monarch and minister drugs in Yinqiao powder.

#### 3.4.5. Principal Component Analysis

SPSS17.0 software was used for principal component analysis. As shown in Table S6 and Table 5, a total of 9 principal components were obtained when the cumulative rate was 100.000% according to the eigenvalue and variance contribution rate. The scree plot (Figure 6(c)) also showed that the slope tends to be gentle after extracting the first 9 components, indicating that these 9 principal components contain most of the chemical information of Yinqiao powder and they were the key components affecting the quality of Yinqiao powder.

The identified common peaks were assigned according to the matrix analysis of the first five components. Among them, the first principal components were peak 3 (neochlorogenic acid), peak 7 (chlorogenic acid), peak 12 (liquiritin), peak 17 (isochlorogenic acid A), peak 21 (isochlorogenic acid C), peak 25 (phillyrin), and peak 26 (arctiin). The second principal components were peak 13 (rutin) and peak 15 (cynaroside). The third principal components were peak 14 (forsythoside A) and peak 19 (hesperidin). The abovementioned components were distributed in the first three principal components, indicating that these components were the key components to ensure the quality of Yinqiao powder, especially the first principal components.

#### 3.4.6. Partial Least Squares Discriminant Analysis

Partial least squares discriminant analysis (PLS-DA) was performed by SIMCA 14.1 software with common peak area (Figure 6(d)). In this model, the VIP value diagram can directly reflect the contribution of each chromatographic peak. The VIP value greater than 1 was usually used as the criterion for screening indicators. The VIP values of peak 14 (forsythoside A), peak 26, peak 7 (chlorogenic acid), peak 10, peak 2, peak 17 (isochlorogenic acid A), peak 12 (liquiritin), and peak 21 (isochlorogenic acid C) were greater than 1. It was speculated that these components were the key components affecting the quality of Yinqiao powder and played important roles in the quality control.

### 3.5. Quantitative Analysis by QAMS

In quantitative analysis of Yinqiao powder, the 7 reference standards were selected according to the results of UPLC-Q-Exactive-Orbitrap-MS and network pharmacology. Although the results of the network pharmacology showed that kaempferol, luteolin, quercetin, etc., may also be the possible components of Yinqiao powder for the treatment of COVID-19, the peak area was relatively small according to the UPLC-Q-Exactive-Orbitrap-MS results. So, it was indicated that the content of these components in Yinqiao powder was low, especially kaempferol, it was not detected in the extract solution of Yinqiao powder.

#### 3.5.1. Investigation of System Applicability and Specificity

**Figure 7:**
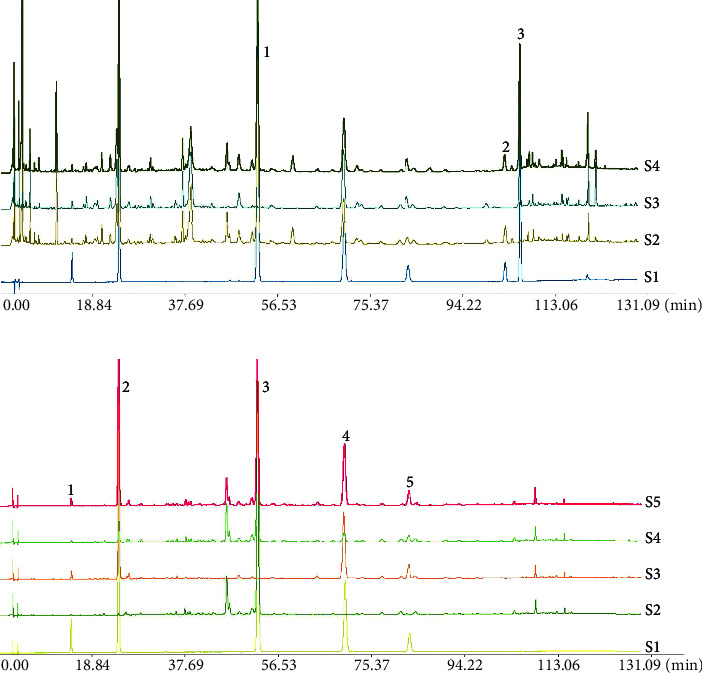
Chromatograms of *yinqiao powder* and negative control samples at 237 nm (a) and 327 nm (b). In panel *a*, 1. forsythoside *A* 2. phillyrin, 3. arctiin, *S*1. mixed reference, *S*2. negative control sample of *Arctii Fructus*, *S*3. negative control sample of *Forsythiae Fructus*, S4. Yinqiao powder. In panel *b*, 1. neochlorogenic acid, 2. chlorogenic acid, 3. forsythoside *A*, 4. isochlorogenic acid *A*, 5. isochlorogenic acid *C*, *S*1. mixed reference, *S*2. negative control sample of *Lonicerae Japonicaeflos* and *Arctii Fructus*, S3. negative control sample of *Forsythiae Fructus*, S4. negative control sample of *Lonicerae japonicaeflos*, S5. Yinqiao powder.

The chromatograms of Yinqiao powder samples and negative samples at 237 nm and 327 nm are shown in Figure 7.

#### 3.5.2. Linearity

Two kinds of mixed reference solutions were analyzed, respectively, and the calibration curves were plotted with different density solution (*X*, *μ*g) and corresponding peak area (*Y*, *A*). The results (Table 6) showed that there was a good correlation between the injection volume and peak area for each compound.

#### 3.5.3. Precision, Stability, Repeatability, and Accuracy

The precision was measured by continuous injection of two kinds of mixed reference solutions for six times at different wavelengths. The stability was validated by analyzing the sample solution of Yinqiao powder at 0, 3, 6, 9, 12, and 24 h at room temperature. Six identical samples of Yinqiao powder were prepared accurately and peak area at different wavelengths were determined in these parallel samples. The accuracy was determined through adding a certain amount of reference substances to six identical samples of Yinqiao powder with a known content. The recovery rate was calculated using the equation (1):

(1) $$\begin{array}{c} \text{Recovery rate }\left(\text{\%}\right)\;=\;\frac{\text{Detected amount}-\text{Original amount}}{\text{Spiked amount}}\times 100\text{\%}. \end{array}$$

As shown in Table S7, the RSD of each component was 0.11%∼2.72% and the recovery rate of seven compounds was 96.32%∼101.79%, indicating that this method could accurately determine the content of seven components in the sample.

#### 3.5.4. Calculation of Relative Correction Factors (RCFs, ƒ_*x*_)

RCFs were calculated using the equation (2):

(2) $$\begin{array}{c} f_{x}=\frac{W_{\text{Forsythoside A}}\times A_{\text{m}}}{W_{\text{m}}\times A_{\text{Forsythoside A}}}. \end{array}$$

In this equation, *W*_*m*_ indicates the concentration of a specific reference substance, and *A*_*m*_ indicates the peak area of reference substance. Forsythoside *A* was selected as the internal standard reference to calculate the RCFs of phillyrin and arctiin at 237 nm, and the RCFs of neochlorogenic acid, chlorogenic acid, isochlorogenic acid A and isochlorogenic acid C were calculated at 327 nm (Table 7).

#### 3.5.5. Reproducibility Investigation of RCFs

The reproducibility of RCFs was investigated under the condition of different instruments and different chromatographic columns. The result showed that the RCFs had a good reproducibility in different instruments and columns (Table S8).

#### 3.5.6. Comparison between QAMS and ESM

The relative errors (REs) built by calculating deviations between the QAMS and ESM ranged from −0.83% to 1.08%. The result showed that there was no significant difference on RE of the two methods, indicating that QAMS was feasible to control the quality of Yinqiao powder (Tables 8 and 9).

## 4. Conclusions

In this study, network pharmacology and UPLC-Q-Exactive-Orbitrap-MS were combined with HPLC fingerprints and QAMS for the quality evaluation of Yinqiao powder for the first time. This quality evaluation method covered four aspects: chemical compositions, pharmacological effects, qualitative analysis, and quantitative analysis.

Network pharmacology and UPLC-Q-Exactive-Orbitrap-MS were used to screen the potential active components of Yinqiao powder. The network pharmacology results showed that Yinqiao powder may inhibit the inflammatory response by suppressing IL-6, CXCL2, TNF*α*, NF-*κ*B, etc., in the treatment of COVID-19. A total of 53 compounds were identified in Yinqiao powder extract and 33 blood components were identified in rat serum using UPLC-Q-Exactive-Orbitrap-MS. The combination of network pharmacology and serum pharmacochemistry could lay the foundation of the medicinal material for Yinqiao powder. In the HPLC fingerprint analysis of Yinqiao powder, 11 compounds were identified. It was clear that the main components were from *Lonicerae Japonicae Flos*, *Forsythiae Fructus*, and *Arctii Fructus* by comparing the chromatograms of single herbs. The multivariate statistical analysis results showed that chlorogenic acid, neochlorogenic acid, isochlorogenic acid A, isochlorogenic acid C, arctiin, phillyrin, and forsythoside A were key components affecting the quality of Yinqiao powder. For the quantitative analysis of Yinqiao powder, the QAMS method was carried out at double-wavelength (237 nm, 327 nm). The content of forsythoside A, phillyrin, arctiin, neochlorogenic acid, chlorogenic acid, isochlorogenic acid A, and isochlorogenic acid C was simultaneously determined using forsythoside A as the internal standard reference. The result of reproducibility investigation of RCFs showed that the reproducibility was good and the RSDs were all less than 3%. In order to verify the reliability and accuracy of the QAMS method established in this experiment, the experimental results measured by QAMS method were compared with those measured by ESM. The comparison results of QAMS and ESM showed that there was no significant difference between the two methods, which indicated that the QAMS method was economical and efficient.

This study did not conduct a more in-depth exploration due to the limited time. In conclusion, the quality evaluation method established in our study could comprehensively and scientifically reflect the stability of the internal quality of Yinqiao powder, which is conducive to the improvement of the quality standard of Yinqiao powder and provides a beneficial guarantee for the clinical treatment of COVID-19.

## Figures and Tables

**Table 1:** Information of the herbal slices in Yinqiao powder.

Herbal slice name	Original plant name	Collection location	Lot number
*Lonicerae Japonicae Flos*	*Lonicera japonica* Thunb.	Shandong	20200108
*Forsythiae Fructus*	*Forsythia suspensa* (Thunb.)	Shanxi	20200101
*Platycodonis Radix*	*Platycodon grandiflorum* (Jacq.) A.DC.	Guizhou	191201
*Menthae Haplocalycis Herba*	*Mentha haplocalyx* Briq.	Jiangsu	20190822
*Sojae Semen Praeparatum*	*Glycine max* (L.) Merr.	Hebei	20200525
*Lophatheri Herba*	*Lophatherum gracile* Brongn.	Zhejiang	20191127
*Arctii Fructus*	*Arctium lappa* L.	Liaoning	180901
*Schizonepetae Spica*	*Schizonepeta tenuifolia* Briq.	Hebei	20190304
*Phragmitis Rhizoma*	*Phragmites communis* Trin.	Hebei	349190801
*Glycyrrhizae Radix et Rhizoma*	*Glycyrrhiza uralensis* Fisch.	Gansu	20200501

**Table 2:** Mass spectrometry information of reference substances.

Reference substance	*t*_R_ (min)	Ion mode	Theoretical (*m/z*)	Measured (*m/z*)	Fragment ions (*m/z*)
Neochlorogenic acid	5.80	[M − H]^−^	353.0867	353.0876	191.0554, 179.0341, 135.0439
Chlorogenic acid	6.93	[M − H]^−^	353.0867	353.0876	191.0554, 179.0341, 135.0439
Caffeic acid	7.34	[M − H]^−^	179.0339	179.0341	135.0439
Liquiritin	10.00	[M − H]^−^	417.1180	417.1196	255.0662, 135.0074, 119.0488
Rutin	10.02	[M − H]^−^	609.1450	609.1464	300.0279, 271.0250, 255.0299
Cynaroside	10.41	[M − H]^−^	447.0922	447.0936	285.0407
Forsythoside *A*	10.93	[M − H]^−^	623.1970	623.1980	461.1674, 161.0232
Isochlorogenic acid *A*	11.80	[M − H]^−^	515.1184	515.1195	353.0882, 191.0554, 179.0446, 135.0439
Isochlorogenic acid *C*	11.83	[M − H]^−^	515.1184	515.1195	353.0882, 191.0554, 179.0446, 135.0439
Hesperidin	11.54	[M − H]^−^	609.1814	609.1813	301.0721
Phillyrin	13.05	[M + HCOO]^−^	579.2072	579.2091	371.1507
Arctiin	13.34	[M + HCOO]^−^	579.2072	579.2091	371.1504

**Table 3:** Information of identified compounds in Yinqiao powder extract.

No	*t*_*R*_ (min)	Name	Formula	Ion mode	Theoretical (*m/z*)	Measured (*m/z*)	ppm	Fragments ion (*m/z*)	Peak area	Reference
1	0.64	Arginine	C_6_H_14_N_4_O_2_	[M + H]^+^	175.1190	175.1191	0.615	175.1191, 116.0709, 70.0659	9.26*E + *08	[22]
2	0.72	Histidine	C_6_H_9_N_3_O_2_	[M + H]^+^	156.0768	156.0768	0.557	138.0549, 110.0717	3.29*E + *07	–
3	0.72	Quinic acid	C_7_H_12_O_6_	[M − H]^−^	191.0550	191.0552	1.180	127.0338, 85.0279	4.62*E + *09	[23]
4	0.73	Proline	C_5_H_9_NO_2_	[M + H]^+^	116.0706	116.0710	2.971	70.0656	5.20*E + *09	[24]
5	0.73	Valine	C_5_H_11_NO_2_	[M + H]^+^	118.0863	118.0866	3.090	100.1125, 72.0816	1.41*E + *09	[22]
6	0.77	Glutamic acid	C_5_H_9_NO_4_	[M + H]^+^	148.0604	148.0605	0.241	148.0605, 130.0500, 102.0554, 84.0451	8.99*E + *07	[25]
7	1.02	Adenine	C_5_H_5_N_5_	[M + H]^+^	136.0618	136.0619	0.869	119.0495, 91.0549	2.17*E + *08	[26]
8	1.19	Isoleucine	C_6_H_13_NO_2_	[M + H]^+^	132.1019	132.1021	1.096	132.1021, 86.0971	4.87*E + *08	[25]
9	1.24	Tyrosine	C_9_H_11_NO_3_	[M + H]^+^	182.0812	182.0813	0.935	165.0546, 136.0757, 123.0443	2.02*E + *08	[24]
10	2.32	Phenylalanine	C_9_H_11_NO_2_	[M + H]^+^	166.0863	166.0864	0.872	120.0810, 107.0496	7.23*E + *07	[26]
11	5.51	Loganic acid	C_16_H_24_O_10_	[M − H]^−^	375.1286	375.1299	3.403	213.0763, 151.0752, 125.0594	3.35*E + *08	[23]
12^*∗*^	5.61	Neochlorogenic acid	C_16_H_18_O_9_	[M − H]^−^	353.0867	353.0882	4.139	191.0553, 179.0340, 135.0438	8.78*E + *08	[24]
13	5.69	Loganin	C_17_H_26_O_10_	[M − H]^−^	389.1442	389.1458	3.923	345.1195, 227.0565, 183.0655, 139.0388	6.16*E + *04	[27]
14	5.84	Forsythoside E	C_20_H_30_O_12_	[M + H]^+^	463.1810	463.1809	−0.265	155.0704, 137.0596	1.72*E + *07	[28]
15	5.92	Methyl cinnamate	C_10_H_10_O_2_	[M + H]^+^	163.0754	163.0754	0.392	135.0442, 131.0493, 103.0547	1.69*E + *07	[25]
16	7.10	Sweroside	C_16_H_22_O_9_	[M − H]^−^	357.1180	357.1195	4.288	195.0661, 177.0545	5.87*E + *05	[29]
17^*∗*^	7.31	Chlorogenic acid	C_16_H_18_O_9_	[M − H]^−^	353.0867	353.0880	1.331	191.0553, 179.0431, 135.0438	1.38*E + *09	[30]
18	7.80	Secoxyloganin	C_17_H_24_O_11_	[M + H]^+^	405.1391	405.1392	0.054	243.0862, 225.0757, 165.0547, 151.0390	1.72*E + *08	[23]
19	7.80	Scopoletin	C_10_H_8_O_4_	[M + H]^+^	193.0495	193.0497	0.698	151.0396, 95.0497	8.68*E + *07	[31]
20	7.97	Glycitin	C_22_H_22_O_10_	[M + H]^+^	447.1286	447.1285	−0.142	285.0756, 270.0522, 242.0572	5.36*E + *07	[32]
21	8.55	Rutin	C_27_H_30_O_16_	[M + H]^+^	611.1607	611.1605	−0.329	465.1021, 303.0498	2.11*E + *08	[23]
22	8.60	Liquiritigenin	C_15_H_12_O_4_	[M + H]^+^	257.0808	257.0806	−1.032	147.0441, 137.0235, 119.0495	1.57*E + *09	[33]
23	8.80	Isoquercitrin	C_21_H_20_O_12_	[M + H]^+^	465.1028	465.1029	0.403	303.1497	4.34*E + *07	[34]
24^*∗*^	8.80	Quercetin	C_15_H_10_O_7_	[M + H]^+^	303.0499	303.0498	−0.426	153.0183, 137.0234	2.02*E + *07	[34]
25	8.85	Cynaroside	C_21_H_20_O_11_	[M + H]^+^	449.1078	449.1078	−0.040	287.0549, 241.0501	9.26*E + *07	[23]
26	8.93	Genistin	C_21_H_20_O_10_	[M + H]^+^	433.1129	433.1129	0.062	271.0598, 243.0652, 215.0702	8.08*E + *07	[32]
27	9.13	Isoschaftoside	C_26_H_28_O_14_	[M − H]^−^	563.1395	563.1411	2.802	383.0769, 353.0667, 297.0754	3.46*E + *07	—
28	9.22	(+)-Pinoresinol	C_20_H_22_O_6_	[M + H]^+^	359.1489	359.1487	−0.654	175.0755, 137.0598	2.20*E + *07	[35]
29	9.23	Pµlegone	C_10_H_16_O	[M + H]^+^	153.1274	153.1274	0.119	109.1016, 81.0706	2.98*E + *07	—
30	9.43	Daidzin	C_21_H_20_O_9_	[M − H]^−^	415.1024	415.1041	1.741	253.0506, 135.0074	3.76*E + *06	[32]
31	9.70	Hesperidin	C_28_H_34_O_15_	[M + H]^+^	611.1970	611.1971	0.153	449.1436, 303.0860, 195.0288	1.64*E + *08	[24]
32	9.83	Forsythoside I	C_29_H_36_O_15_	[M − H]^−^	623.1970	623.1985	2.316	461.1655, 161.0233	2.53*E + *09	[28]
33^*∗*^	10.00	Liquiritin	C_21_H_22_O_9_	[M − H]^−^	417.1180	417.1195	3.527	255.0663, 135.0074, 119.0488	1.29*E + *09	[33]
34^*∗*^	10.26	Forsythoside A	C_29_H_36_O_15_	[M − H]^−^	623.1970	623.1985	2.316	461.1674, 161.0232	3.38*E + *09	[28]
35	10.45	Lonicerin	C_27_H_30_O_15_	[M − H]^−^	593.1501	593.1520	3.175	447.0936, 285.0406	3.51*E + *07	[23]
36	10.51	Ononin	C_22_H_22_O_9_	[M + H]^+^	431.1337	431.1335	−0.345	269.0806, 254.0572	2.98*E + *08	[36]
37	10.60	Calceolarioside B	C_23_H_26_O_11_	[M − H]^−^	477.1391	477.1407	3.253	161.0232, 133.0281	3.76*E + *08	[36]
38	10.83	Kaempferol-3-O-rutinoside	C_27_H_30_O_15_	[M − H]^−^	593.1501	593.1517	2.754	285.0407, 255.0298, 227.0345	3.70*E + *07	[23]
39	10.91	Daidzein	C_15_H_10_O_4_	[M + H]^+^	255.0652	255.0650	−0.766	227.0701, 199.0754, 137.0234	4.22*E + *08	[32]
40	11.03	Isochlorogenic acid B	C_25_H_24_O_12_	[M − H]^−^	515.1184	515.1180	−0.704	353.0877, 191.0552, 179.0340, 173.0445, 135.0437	6.27*E + *08	[23]
41	11.09	Arctigenin	C_21_H_24_O_6_	[M + H]^+^	373.1646	373.1642	−0.924	237.1121, 137.0597	2.93*E + *09	[21]
42	11.17	Linarin	C_28_H_32_O_14_	[M + H]^+^	593.1865	593.1863	−0.341	447.1284, 285.0756, 270.0523, 242.0571	7.65*E + *07	—
43	11.22	Glycitein	C_16_H_12_O_5_	[M + H]^+^	285.0758	285.0757	−0.351	270.0522, 242.0573	1.10*E + *08	[32]
44^*∗*^	11.23	Isochlorogenic acid A	C_25_H_24_O_12_	[M − H]^−^	515.1184	515.1189	0.966	353.0881, 191.0554, 179.0341, 173.0445, 135.0438	4.17*E + *08	[23]
45	11.46	Azelaic acid	C_9_H_16_O_4_	[M − H]^−^	187.0965	187.0968	1.681	143.1061, 125.0958, 97.0643	2.03*E + *08	—
46^*∗*^	11.76	Isochlorogenic acid C	C_25_H_24_O_12_	[M − H]^−^	515.1184	515.1171	−2.470	353.0869, 191.0557, 179.0340, 135.0439	8.75*E + *08	[23]
47	11.79	Ferµlic acid	C_10_H_10_O_4_	[M − H]^−^	193.0495	193.0499	2.045	178.0261, 161.0232, 133.0281	6.04*E + *07	[37]
48	12.52	Isoliquiritin	C_21_H_22_O_9_	[M − H]^−^	417.1180	417.1197	3.959	255.0661, 180.0054, 135.0074, 119.0487	2.29*E + *08	[33]
49	12.67	Genistein	C_15_H_10_O_5_	[M + H]^+^	271.0601	271.0600	−0.258	243.0649, 215.0704, 153.0183	7.06*E + *07	[32]
50^*∗*^	13.04	Phillyrin	C_27_H_34_O_11_	[*M* + HCOO]^−^	579.2072	579.2088	2.732	371.1496, 356.1265	1.03*E + *09	[21]
51^*∗*^	13.32	Arctiin	C_27_H_34_O_11_	[*M* + HCOO]^−^	579.2072	579.2087	2.525	371.1500, 356.1268	5.10*E + *09	[21]
52^*∗*^	13.69	Luteolin	C_15_H_10_O_6_	[M − H]^−^	285.0394	285.0406	4.194	151.0025, 133.0282, 107.0124	6.19*E + *04	[33]
53	15.37	Matairesinol	C_20_H_22_O_6_	[M − H]^−^	357.1332	357.1347	3.935	83.0122	2.13*E + *08	—

^*∗*^: components with potential pharmacological effects; —: compared with Chinese medicine database.

**Table 4:** Information of blood components in Yinqiao powder.

Number	Name	Extract	Drug serum	Blank serum
1	Ferulic acid	√	√	—
2	Daidzein	√	√	—
3	Genistein	√	√	—
4	Liquiritigenin	√	√	—
5	Sweroside	√	√	—
6	Loganic acid	√	√	—
7	Secoxyloganin	√	√	—
8	Loganin	√	√	—
9^*∗*^	Liquiritin	√	√	—
10	Ononin	√	√	—
11^*∗*^	Chlorogenic acid	√	√	—
12	Forsythoside *E*	√	√	—
13^*∗*^	Isochlorogenic acid *C*	√	√	—
14^*∗*^	Isochlorogenic acid *A*	√	√	—
15^*∗*^	Neochlorogenic acid	√	√	—
16	Glycitein	√	√	—
17	Genistin	√	√	—
18	Arctigenin	√	√	—
19^*∗*^	Arctiin	√	√	—
20	Pµlegone	√	√	—
21^*∗*^	Forsythoside *A*	√	√	—
22	Daidzein	√	√	—
23^*∗*^	Phillyrin	√	√	—
24	Apigenin 7-O-glucuronide	—	√	—
25	Geniposidic acid	—	√	—
26	Hexadecanedioic acid	—	√	—
27	OroxylinA-7-O-*β*-D-glucuronide	—	√	—
28	Naringin	—	√	—
29	Glutathione	—	√	—
30	Eucalyptol	—	√	—
31	Formononetin	—	√	—
32	Sinapine	—	√	—
33	18-*β*-Glycyrrhetinic acid	—	√	—

^*∗*^: components with potential pharmacological effects; √: existence, —: nonexistence.

**Table 5:** Initial factor load matrix analysis of common peaks.

Common peak no.	*Component*				
	1	2	3	4	5
1	0.480	0.621	0.115	−0.489	−0.122
2	0.941	−0.240	0.220	−0.021	0.070
3	0.991	0.034	0.108	−0.029	−0.019
4	0.799	−0.155	−0.244	0.378	0.330
5	−0.341	0.921	−0.133	−0.008	0.095
6	−0.221	0.668	−0.438	0.316	0.331
7	0.764	−0.462	0.319	−0.267	−0.125
8	0.867	0.474	0.022	0.063	0.008
9	0.969	−0.178	0.123	−0.060	0.041
10	0.742	−0.624	0.189	−0.093	0.030
11	−0.438	0.567	0.594	−0.222	−0.219
12	0.919	0.284	0.138	−0.202	−0.013
13	−0.448	0.750	0.433	0.183	0.081
14	−0.172	−0.133	0.730	0.598	−0.083
15	0.201	0.644	−0.105	0.408	−0.454
16	−0.511	−0.232	0.688	0.418	0.107
17	0.898	0.356	0.067	−0.143	0.147
18	0.483	−0.846	−0.043	0.111	−0.177
19	−0.623	−0.697	−0.148	0.242	0.106
20	−0.730	−0.380	0.173	−0.456	0.091
21	0.959	−0.076	0.001	0.096	−0.185
22	0.015	−0.522	0.287	−0.283	0.619
23	0.548	0.794	0.037	0.162	0.174
24	−0.931	0.104	0.313	−0.016	0.039
25	0.495	−0.680	0.408	0.346	−0.021
26	0.935	0.287	0.061	−0.173	0.041
27	0.520	0.750	0.171	0.283	0.219
28	0.949	0.018	0.014	0.236	0.036
29	0.178	−0.605	−0.623	0.275	−0.093

**Table 6:** Linear regression equation of seven reference substances.

Wavelength (nm)	Reference substance	Regression equations	*R*	Linear range (*μ*g)
237	Forsythoside *A*	*y* = 26.30*x* + 0.073	0.9995	0.1776–2.96
	Phillyrin	*y* = 21.89*x* + 0.024	0.9995	0.0165–0.275
	Arctiin	*y* = 22.68*x* + 0.073	0.9995	0.0678–1.130

327	Forsythoside *A*	*y* = 43.42*x* + 0.065	0.9995	0.1776–2.960
	Neochlorogenic acid	*y* = 94.10*x* + 0.048	0.9995	0.00462–0.077
	Chlorogenic acid	*y* = 70.02*x* + 0.027	0.9995	0.0576–0.960
	Isochlorogenic acid *A*	*y* = 68.79*x* − 0.420	0.9995	0.039–0.650
	Isochlorogenic acid *C*	*y* = 78.88*x* − 0.090	0.9995	0.00912–0.152

**Table 7:** The RCFs of components at 237 nm and 327 nm.

	237 nm		327 nm			
	Phillyrin	Arctiin	Neochlorogenic acid	Chlorogenic acid	Isochlorogenic acid *A*	Isochlorogenic acid *C*
1	0.832	0.867	2.179	1.613	1.567	1.793
2	0.836	0.869	2.185	1.614	1.567	1.800
3	0.838	0.867	2.178	1.613	1.566	1.796
4	0.841	0.867	2.181	1.611	1.564	1.803
5	0.840	0.869	2.181	1.614	1.566	1.802
6	0.838	0.867	2.187	1.614	1.566	1.795
Mean	0.838	0.868	2.182	1.613	1.566	1.798
RSD	0.41%	0.13%	0.17%	0.06%	0.06%	0.23%

**Table 8:** The calculation results of the ESM and QAMS method at 237 nm.

Batch	Forsythoside A	Phillyrin			Arctiin		
	ESM	ESM	QAMS	RE %	ESM	QAMS	RE %
1	8.689	0.868	0.862	−0.69	5.077	5.062	−0.30
2	9.311	1.292	1.283	−0.70	4.763	4.748	−0.31
3	8.260	1.146	1.138	−0.70	6.034	6.016	−0.30
4	8.193	1.107	1.099	−0.72	6.024	6.005	−0.32
5	8.224	1.043	1.036	−0.67	6.195	6.176	−0.31
6	9.462	1.125	1.117	−0.71	5.878	5.860	−0.31
7	9.346	1.110	1.103	−0.63	5.653	5.635	−0.32
8	9.316	1.229	1.220	−0.73	5.445	5.428	−0.31
9	8.376	0.993	0.987	−0.60	4.713	4.699	−0.30
10	8.653	1.114	1.107	−0.63	6.257	6.238	−0.30

**Table 9:** The calculation results of the ESM and QAMS method at 327 nm.

Batch	Forsythoside *A*	Neochlorogenic acid			Chlorogenic acid			Isochlorogenic acid *A*			Isochlorogenic acid *C*		
	ESM	ESM	QAMS	RE %	ESM	QAMS	RE %	ESM	QAMS	RE %	ESM	QAMS	RE %
1	8.603	0.063	0.063	0.00	3.633	3.629	−0.11	2.502	2.502	0.00	0.436	0.433	−0.69
2	9.202	0.070	0.070	0.00	4.283	4.279	−0.09	2.349	2.349	0.00	0.540	0.536	−0.74
3	8.165	0.095	0.095	0.00	4.438	4.433	−0.11	2.871	2.872	0.03	0.669	0.665	−0.60
4	8.082	0.094	0.094	0.00	4.590	4.585	−0.11	2.760	2.761	0.04	0.685	0.680	−0.73
5	8.133	0.093	0.094	1.08	4.360	4.355	−0.11	2.828	2.828	0.00	0.691	0.687	−0.58
6	9.424	0.087	0.087	0.00	4.305	4.301	−0.09	2.792	2.792	0.00	0.616	0.612	−0.65
7	9.300	0.086	0.086	0.00	4.198	4.194	−0.10	2.753	2.753	0.00	0.604	0.599	−0.83
8	9.234	0.086	0.086	0.00	4.389	4.384	−0.11	2.589	2.590	0.04	0.642	0.638	−0.62
9	8.317	0.064	0.064	0.00	3.816	3.812	−0.10	2.342	2.343	0.04	0.485	0.482	−0.62
10	8.549	0.096	0.096	0.00	4.394	4.389	−0.11	2.826	2.826	0.00	0.707	0.703	−0.57

## Data Availability

The data used to support the findings of this study are included within the article and supplementary information file.

## Abbreviations

HPLC: High-performance liquid chromatography
UPLC: Ultrahigh-performance liquid chromatography
QAMS: Quantitative analysis of multi-components by single marker
ESM: External standard method
COVID-19: Coronavirus disease 2019
TCM: Traditional Chinese medicine
SARS: Severe acute respiratory syndrome
OB: Oral bioavailability
DL: Drug-likeness
Ch. P: Pharmacopoeia of the People's Republic of China
PPI: Protein-protein interaction
GO: Gene Ontology
KEGG: Kyoto Encyclopedia of Genes and Genomes
BP: Biological processes
CC: Cellular components
MF: Molecular functions
NCE: Normalized collision energy
UAE: Ultrasonic assisted extraction
SNR: Signal-to-noise ratio
TIC: Total ion chromatogram
RSD: Relative standard deviation
PCA: Principal component analysis
PLS-DA: Partial least squares discriminant analysis
RCF: Relative correction factor
RE: Relative error.

## Supplementary Materials

Supplementary Materials Table S1: Information of the standard materials; Table S2: The KEGG enrichment results of Yinqiao powder in treating for COVID-19; Table S3: Similarity evaluation of 10 batches of Yinqiao powder; Table S4: Relative retention time and RSD of common peaks; Table S5: Median retention time and average peak area of common peaks; Table S6: Variance contribution rates of principal components; Table S7: Results of method validation; Table S8: RCFs in different instruments and columns.
