# Supplementary material for: Establishment of Quality Evaluation Method for Yinqiao Powder: A Herbal Formula against COVID-19 in China
Source: J Anal Methods Chem. 2022 Nov 25;2022:1748324. doi: 10.1155/2022/1748324 (PMC9718632; doi:10.1155/2022/1748324)
Supplement: Supplementary Materials — Table S1: Information of the standard materials; Table S2: The KEGG enrichment results of Yinqiao powder in treating for COVID-19; Table S3: Similarity evaluation of 10 batches of Yinqiao powder; Table S4: Relative retention time and RSD of common peaks; Table S5: Median retention time and average peak area of common peaks; Table S6: Variance contribution rates of principal components; Table S7: Results of method validation; Table S8: RCFs in different instruments and columns. [file 1748324.f1.pdf]

Supplementary Materials

Table S1. Information of the standard materials.

Standard Materials	Lot Number	Content
Caffeic acid	110885-200102	97.2%
Phillyrin	Z17A8X34077	98%
Arctiin	Z11D6B7394	98%
Neochlorogenic acid	P13O8F45676	98%
Chlorogenic acid	Y24J7K16726	98%
Isochlorogenic acid A	Y24N8Y49009	98%
Isochlorogenic acid C	P25J6F1794	98%
Hesperidin	110721-201014	95.1%
Rutin	100080-201409	91.9%
Liquiritin	111610-201106	93.7%
Cynaroside	111720-201408	94.9%
Forsythoside A	111810-201707	97.2%

Table S2. The KEGG enrichment results of Yinqiao powder in treating for COVID-19.

GO	Description	Count	%	LogP
hsa05200	Pathways in cancer	40	50.00	-48.09
hsa05417	Lipid and atherosclerosis	31	38.75	-45.52
hsa04933	AGE-RAGE signaling pathway in diabetic complications	23	28.75	-38.45
hsa05163	Human cytomegalovirus infection	24	30.00	-31.56
hsa05167	Kaposi sarcoma-associated herpesvirus infection	22	27.50	-29.47
hsa04657	IL-17 signaling pathway	18	22.50	-28.39
hsa05205	Proteoglycans in cancer	21	26.25	-27.14
hsa05418	Fluid shear stress and atherosclerosis	19	23.75	-27.00
hsa04668	TNF signaling pathway	18	22.50	-26.91
hsa05215	Prostate cancer	17	21.25	-26.09
hsa05165	Human papillomavirus infection	23	28.75	-25.83
hsa05161	Hepatitis B	19	23.75	-25.67
hsa05145	Toxoplasmosis	17	21.25	-24.95
hsa05171	Coronavirus disease - COVID-19	20	25.00	-24.28
hsa05142	Chagas disease	16	20.00	-23.72
hsa04151	PI3K-Akt signaling pathway	22	27.50	-23.60
hsa04066	HIF-1 signaling pathway	16	20.00	-23.23
hsa05164	Influenza A	17	21.25	-21.68
hsa01521	EGFR tyrosine kinase inhibitor resistance	14	17.50	-21.56
hsa05162	Measles	16	20.00	-21.46
hsa05166	Human T-cell leukemia virus 1 infection	18	22.50	-21.33
hsa05160	Hepatitis C	16	20.00	-20.58
hsa05169	Epstein-Barr virus infection	17	21.25	-20.42
hsa05206	MicroRNAs in cancer	19	23.75	-20.21
hsa01522	Endocrine resistance	14	17.50	-20.16
hsa05225	Hepatocellular carcinoma	16	20.00	-20.09

hsa05207	Chemical carcinogenesis - receptor activation	17	21.25	-20.06
hsa05212	Pancreatic cancer	13	16.25	-19.82
hsa05222	Small cell lung cancer	13	16.25	-18.67
hsa04210	Apoptosis	14	17.50	-18.08
hsa04620	Toll-like receptor signaling pathway	13	16.25	-17.94
hsa04625	C-type lectin receptor signaling pathway	13	16.25	-17.94
hsa04936	Alcoholic liver disease	14	17.50	-17.81
hsa04010	MAPK signaling pathway	17	21.25	-17.63
hsa04218	Cellular senescence	14	17.50	-17.22
hsa05210	Colorectal cancer	12	15.00	-17.18
hsa04071	Sphingolipid signaling pathway	13	16.25	-17.15
hsa04919	Thyroid hormone signaling pathway	13	16.25	-17.05
hsa05170	Human immunodeficiency virus 1 infection	15	18.75	-16.87
hsa05022	Pathways of neurodegeneration - multiple diseases	19	23.75	-16.72
hsa04926	Relaxin signaling pathway	13	16.25	-16.68
hsa04068	FoxO signaling pathway	13	16.25	-16.59
hsa05208	Chemical carcinogenesis - reactive oxygen species	15	18.75	-16.54
hsa04917	Prolactin signaling pathway	11	13.75	-16.38
hsa05152	Tuberculosis	14	17.50	-16.34
hsa05135	Yersinia infection	13	16.25	-16.33
hsa05146	Amoebiasis	12	15.00	-16.25
hsa04621	NOD-like receptor signaling pathway	14	17.50	-16.20
hsa01524	Platinum drug resistance	11	13.75	-16.16
hsa04115	p53 signaling pathway	11	13.75	-16.16
hsa04064	NF-kappa B signaling pathway	12	15.00	-16.14
hsa05214	Glioma	11	13.75	-16.03
hsa05220	Chronic myeloid leukemia	11	13.75	-15.96
hsa04062	Chemokine signaling pathway	14	17.50	-15.94
hsa04659	Th17 cell differentiation	12	15.00	-15.94
hsa05140	Leishmaniasis	11	13.75	-15.89
hsa05131	Shigellosis	15	18.75	-15.88
hsa05132	Salmonella infection	15	18.75	-15.83
hsa04510	Focal adhesion	14	17.50	-15.66
hsa04932	Non-alcoholic fatty liver disease	13	16.25	-15.62
hsa04370	VEGF signaling pathway	10	12.50	-15.27
hsa05235	PD-L1 expression and PD-1 checkpoint pathway in cancer	11	13.75	-15.17
hsa04380	Osteoclast differentiation	12	15.00	-15.03
hsa05323	Rheumatoid arthritis	11	13.75	-14.95
hsa05219	Bladder cancer	9	11.25	-14.88
hsa05223	Non-small cell lung cancer	10	12.50	-14.35
hsa05130	Pathogenic Escherichia coli infection	13	16.25	-14.25
hsa05133	Pertussis	10	12.50	-14.10
hsa04935	Growth hormone synthesis, secretion and action	11	13.75	-13.73
hsa05213	Endometrial cancer	9	11.25	-13.41
hsa05202	Transcriptional misregulation in cancer	12	15.00	-12.87
hsa05224	Breast cancer	11	13.75	-12.71
hsa04660	T cell receptor signaling pathway	10	12.50	-12.69
hsa05226	Gastric cancer	11	13.75	-12.64
hsa05203	Viral carcinogenesis	12	15.00	-12.58
hsa05218	Melanoma	9	11.25	-12.52
hsa05020	Prion disease	13	16.25	-12.43
hsa04915	Estrogen signaling pathway	10	12.50	-11.45
hsa04371	Apelin signaling pathway	10	12.50	-11.41

hsa04921	Oxytocin signaling pathway	10	12.50	-10.97
hsa04931	Insulin resistance	9	11.25	-10.89
hsa04630	JAK-STAT signaling pathway	10	12.50	-10.75
hsa04726	Serotonergic synapse	9	11.25	-10.64
hsa05010	Alzheimer disease	13	16.25	-10.57
hsa05144	Malaria	7	8.75	-10.20
hsa04012	ErbB signaling pathway	8	10.00	-10.16
hsa05231	Choline metabolism in cancer	8	10.00	-9.66
hsa04215	Apoptosis - multiple species	6	7.50	-9.61
hsa04061	Viral protein interaction with cytokine and cytokine receptor	8	10.00	-9.59
hsa04150	mTOR signaling pathway	9	11.25	-9.45
hsa04928	Parathyroid hormone synthesis, secretion and action	8	10.00	-9.38
hsa04664	Fc epsilon RI signaling pathway	7	8.75	-9.22
hsa04014	Ras signaling pathway	10	12.50	-9.22
hsa05168	Herpes simplex virus 1 infection	13	16.25	-9.21
hsa05216	Thyroid cancer	6	7.50	-9.21
hsa04725	Cholinergic synapse	8	10.00	-9.16
hsa05230	Central carbon metabolism in cancer	7	8.75	-9.13
hsa04722	Neurotrophin signaling pathway	8	10.00	-8.98
hsa04662	B cell receptor signaling pathway	7	8.75	-8.64
hsa05014	Amyotrophic lateral sclerosis	11	13.75	-8.48
hsa05415	Diabetic cardiomyopathy	9	11.25	-8.44
hsa04140	Autophagy-animal	8	10.00	-8.39
hsa04658	Th1 and Th2 cell differentiation	7	8.75	-8.29
hsa04912	GnRH signaling pathway	7	8.75	-8.26
hsa04060	Cytokine-cytokine receptor interaction	10	12.50	-8.21
hsa05134	Legionellosis	6	7.50	-8.03
hsa04217	Necroptosis	8	10.00	-7.98
hsa05416	Viral myocarditis	6	7.50	-7.89
hsa04929	GnRH secretion	6	7.50	-7.72
hsa05321	Inflammatory bowel disease	6	7.50	-7.68
hsa05221	Acute myeloid leukemia	6	7.50	-7.60
hsa05211	Renal cell carcinoma	6	7.50	-7.52
hsa04622	RIG-I-like receptor signaling pathway	6	7.50	-7.49
hsa05120	Epithelial cell signaling in Helicobacter pylori infection	6	7.50	-7.49
hsa04611	Platelet activation	7	8.75	-7.39
hsa04613	Neutrophil extracellular trap formation	8	10.00	-7.38
hsa04110	Cell cycle	7	8.75	-7.34
hsa05143	African trypanosomiasis	5	6.25	-7.33
hsa04650	Natural killer cell mediated cytotoxicity	7	8.75	-7.22
hsa04728	Dopaminergic synapse	7	8.75	-7.20
hsa04015	Rap1 signaling pathway	8	10.00	-7.04
hsa04540	Gap junction	6	7.50	-6.89
hsa04723	Retrograde endocannabinoid signaling	7	8.75	-6.86
hsa04072	Phospholipase D signaling pathway	7	8.75	-6.86
hsa04211	Longevity regulating pathway	6	7.50	-6.86
hsa04261	Adrenergic signaling in cardiomyocytes	7	8.75	-6.82
hsa04934	Cushing syndrome	7	8.75	-6.72
hsa04666	Fc gamma R-mediated phagocytosis	6	7.50	-6.63
hsa04713	Circadian entrainment	6	7.50	-6.63
hsa04916	Melanogenesis	6	7.50	-6.53
hsa04914	Progesterone-mediated oocyte maturation	6	7.50	-6.50
hsa05012	Parkinson disease	8	10.00	-6.26

hsa04720	Long-term potentiation	5	6.25	-6.01
hsa05031	Amphetamine addiction	5	6.25	-5.95
hsa04920	Adipocytokine signaling pathway	5	6.25	-5.95
hsa01523	Antifolate resistance	4	5.00	-5.93
hsa04918	Thyroid hormone synthesis	5	6.25	-5.77
hsa04550	Signaling pathways regulating pluripotency of stem cells	6	7.50	-5.64
hsa04960	Aldosterone-regulated sodium reabsorption	4	5.00	-5.55
hsa04610	Complement and coagulation cascades	5	6.25	-5.50
hsa04020	Calcium signaling pathway	7	8.75	-5.45
hsa04310	Wnt signaling pathway	6	7.50	-5.25
hsa04672	Intestinal immune network for IgA production	4	5.00	-5.05
hsa04961	Endocrine and other factor-regulated calcium reabsorption	4	5.00	-4.92
hsa04670	Leukocyte transendothelial migration	5	6.25	-4.87
hsa04724	Glutamatergic synapse	5	6.25	-4.87
hsa04340	Hedgehog signaling pathway	4	5.00	-4.82
hsa04730	Long-term depression	4	5.00	-4.70
hsa04213	Longevity regulating pathway - multiple species	4	5.00	-4.64
hsa04114	Oocyte meiosis	5	6.25	-4.58
hsa00220	Arginine biosynthesis	3	3.75	-4.58
hsa04270	Vascular smooth muscle contraction	5	6.25	-4.53
hsa04910	Insulin signaling pathway	5	6.25	-4.49
hsa04141	Protein processing in endoplasmic reticulum	5	6.25	-4.03
hsa04350	TGF-beta signaling pathway	4	5.00	-3.93
hsa04750	Inflammatory mediator regulation of TRP channels	4	5.00	-3.86
hsa05330	Allograft rejection	3	3.75	-3.85
hsa05016	Huntington disease	6	7.50	-3.78
hsa05332	Graft-versus-host disease	3	3.75	-3.72
hsa04930	Type II diabetes mellitus	3	3.75	-3.60
hsa04810	Regulation of actin cytoskeleton	5	6.25	-3.54
hsa04152	AMPK signaling pathway	4	5.00	-3.53
hsa04024	cAMP signaling pathway	5	6.25	-3.51
hsa04913	Ovarian steroidogenesis	3	3.75	-3.47
hsa00330	Arginine and proline metabolism	3	3.75	-3.47
hsa04923	Regulation of lipolysis in adipocytes	3	3.75	-3.35
hsa04623	Cytosolic DNA-sensing pathway	3	3.75	-3.20
hsa05217	Basal cell carcinoma	3	3.75	-3.20
hsa04390	Hippo signaling pathway	4	5.00	-3.09
hsa05204	Chemical carcinogenesis - DNA adducts	3	3.75	-3.08
hsa04520	Adherens junction	3	3.75	-3.05
hsa04137	Mitophagy - animal	3	3.75	-3.03
hsa00982	Drug metabolism - cytochrome P450	3	3.75	-3.03
hsa04022	cGMP-PKG signaling pathway	4	5.00	-2.99
hsa04971	Gastric acid secretion	3	3.75	-2.96
hsa04612	Antigen processing and presentation	3	3.75	-2.93
hsa00980	Metabolism of xenobiotics by cytochrome P450	3	3.75	-2.93
hsa00983	Drug metabolism - other enzymes	3	3.75	-2.90
hsa04360	Axon guidance	4	5.00	-2.85
hsa04911	Insulin secretion	3	3.75	-2.81
hsa04727	GABAergic synapse	3	3.75	-2.76
hsa04976	Bile secretion	3	3.75	-2.76
hsa05410	Hypertrophic cardiomyopathy	3	3.75	-2.75
hsa05032	Morphine addiction	3	3.75	-2.74
hsa04970	Salivary secretion	3	3.75	-2.72

hsa05414	Dilated cardiomyopathy	3	3.75	-2.67
hsa04070	Phosphatidylinositol signaling system	3	3.75	-2.66
hsa04925	Aldosterone synthesis and secretion	3	3.75	-2.64
hsa04922	Glucagon signaling pathway	3	3.75	-2.54
hsa05322	Systemic lupus erythematosus	3	3.75	-2.24
hsa05017	Spinocerebellar ataxia	3	3.75	-2.18

Table S3. Similarity evaluation of 10 batches of Yinqiao powder.

	S1	S2	S3	S4	S5	S6	S7	S8	S9	S10	Reference
S1	1.000	0.983	0.976	0.973	0.983	0.993	0.993	0.977	0.994	0.984	0.990
S2	0.983	1.000	0.987	0.986	0.983	0.993	0.993	0.994	0.996	0.987	0.994
S3	0.976	0.987	1.000	0.999	0.996	0.993	0.993	0.992	0.988	0.995	0.996
S4	0.973	0.986	0.999	1.000	0.994	0.991	0.991	0.992	0.986	0.994	0.995
S5	0.983	0.983	0.996	0.994	1.000	0.993	0.993	0.988	0.989	0.998	0.996
S6	0.993	0.993	0.993	0.991	0.993	1.000	1.000	0.993	0.998	0.994	0.999
S7	0.993	0.993	0.993	0.991	0.993	1.000	1.000	0.993	0.998	0.994	0.999
S8	0.977	0.994	0.992	0.992	0.988	0.993	0.993	1.000	0.992	0.990	0.995
S9	0.994	0.996	0.988	0.986	0.989	0.998	0.998	0.992	1.000	0.990	0.997
S10	0.984	0.987	0.995	0.994	0.998	0.994	0.994	0.990	0.990	1.000	0.997
Reference	0.990	0.994	0.996	0.995	0.996	0.999	0.999	0.995	0.997	0.997	1.000

Table S4. Relative retention time and RSD of common peaks.

	S1	S2	S3	S4	S5	S6	S7	S8	S9	S10	RSD/%
1	0.118	0.117	0.117	0.117	0.117	0.117	0.117	0.117	0.117	0.117	0.14
2	0.220	0.220	0.220	0.220	0.219	0.219	0.220	0.219	0.220	0.220	0.05
3	0.281	0.281	0.281	0.281	0.280	0.280	0.280	0.280	0.280	0.281	0.11
4	0.396	0.396	0.395	0.395	0.395	0.395	0.395	0.395	0.395	0.395	0.07
5	0.429	0.429	0.428	0.428	0.428	0.428	0.428	0.428	0.428	0.429	0.08
6	0.454	0.453	0.453	0.453	0.452	0.453	0.453	0.453	0.453	0.453	0.08
7	0.463	0.463	0.463	0.462	0.462	0.462	0.462	0.462	0.462	0.463	0.09
8	0.585	0.583	0.583	0.583	0.583	0.583	0.583	0.583	0.583	0.583	0.10
9	0.710	0.708	0.708	0.708	0.707	0.707	0.708	0.708	0.707	0.707	0.11
10	0.741	0.739	0.739	0.739	0.738	0.739	0.739	0.739	0.738	0.739	0.11
11	0.883	0.881	0.881	0.881	0.881	0.881	0.881	0.881	0.881	0.881	0.09
12	0.929	0.928	0.928	0.927	0.927	0.927	0.927	0.927	0.927	0.928	0.08
13	0.980	0.979	0.980	0.979	0.979	0.979	0.979	0.979	0.980	0.980	0.05
14	1.000	1.000	1.000	1.000	1.000	1.000	1.000	1.000	1.000	1.000	0.00
15	1.055	1.051	1.052	1.052	1.051	1.052	1.052	1.052	1.052	1.053	0.09
16	1.138	1.136	1.136	1.136	1.136	1.136	1.136	1.136	1.136	1.136	0.06
17	1.337	1.335	1.335	1.334	1.334	1.334	1.333	1.333	1.335	1.335	0.08
18	1.385	1.382	1.383	1.383	1.383	1.384	1.384	1.384	1.383	1.383	0.06
19	1.403	1.401	1.401	1.400	1.400	1.400	1.401	1.401	1.400	1.402	0.06

20	1.558	1.556	1.555	1.554	1.554	1.554	1.554	1.554	1.555	1.555	0.08
21	1.585	1.583	1.583	1.582	1.582	1.582	1.582	1.581	1.583	1.582	0.07
22	1.615	1.612	1.612	1.609	1.609	1.607	1.608	1.608	1.610	1.610	0.14
23	1.674	1.669	1.668	1.611	1.611	1.612	1.612	1.612	1.611	1.611	1.76
24	1.735	1.731	1.731	1.732	1.732	1.733	1.734	1.735	1.732	1.733	0.09
25	1.966	1.959	1.959	1.959	1.958	1.960	1.960	1.960	1.958	1.958	0.11
26	2.026	2.019	2.018	2.018	2.017	2.018	2.018	2.018	2.016	2.016	0.14
27	2.063	2.056	2.054	2.054	2.053	2.054	2.054	2.054	2.052	2.053	0.15
28	2.291	2.282	2.280	2.280	2.279	2.280	2.243	2.262	2.259	2.240	0.78
29	2.321	2.312	2.310	2.310	2.308	2.309	2.310	2.310	2.308	2.308	0.17

Table S5. Median retention time and average peak area of common peaks.

NO.	t_R	A	NO.	t_R	A
1	6.152	1.518	16	59.610	2.812
2	11.520	4.579	17	70.031	9.909
3	14.717	0.423	18	72.586	1.023
4	20.736	1.339	19	73.497	0.506
5	22.469	1.612	20	81.585	0.465
6	23.764	3.972	21	83.037	2.748
7	24.259	17.379	22	84.476	0.955
8	30.593	0.913	23	87.582	0.864
9	37.138	2.025	24	90.918	0.745
10	38.776	7.616	25	102.826	2.467
11	46.233	3.457	26	105.913	13.022
12	48.677	2.287	27	107.822	1.252
13	51.391	1.599	28	119.678	2.250
14	52.482	23.556	29	121.231	1.808
15	55.217	0.683			

Table S6. Variance contribution rates of principal components.

**Component**	**Total**	**Variance%**	**Cumulative%**
1	13.667	47.129	47.129
2	7.939	27.375	74.504
3	2.900	9.999	84.503
4	2.170	7.481	91.984
5	1.126	3.884	95.868
6	0.659	2.272	98.140
7	0.333	1.147	99.286
8	0.186	0.643	99.929
9	0.021	0.071	100.000
10	$8.678\ E^{-16}$	$2.992E^{-15}$	100.000

11	7.539 E^{-16}	2.538E^{-15}	100.000
12	5.302 E^{-16}	1.828E^{-15}	100.000
13	4.078 E^{-16}	1.406E^{-15}	100.000
14	2.943 E^{-16}	1.015E^{-15}	100.000
15	2.577 E^{-16}	8.885E^{-16}	100.000
16	1.898 E^{-16}	6.546E^{-16}	100.000
17	1.646 E^{-16}	5.675E^{-16}	100.000
18	9.243 E^{-17}	3.187E^{-16}	100.000
19	3.087 E^{-17}	1.065E^{-16}	100.000
20	2.802 E^{-18}	9.663E^{-18}	100.000
21	-8.187 E^{-17}	-2.823E^{-16}	100.000
22	-1.154 E^{-16}	-3.980E^{-16}	100.000
23	-2.303 E^{-16}	-7.943E^{-16}	100.000
24	-2.586 E^{-16}	-8.917E^{-16}	100.000
25	-2.932 E^{-16}	-1.011E^{-15}	100.000
26	-4.315 E^{-16}	-1.488E^{-15}	100.000
27	-4.957 E^{-16}	-1.709E^{-15}	100.000
28	-6.745 E^{-16}	-2.326E^{-15}	100.000
29	-7.601 E^{-16}	-2.621E^{-15}	100.000

Table S7. Results of method validation.

Methodology \ RSD/%	237 nm			327 nm				
	Forsythoside A	Phillyrin	Arctiin	Forsythoside A	Neochlorogenic acid	Chlorogenic acid	Isochlorogenic acid A	Isochlorogenic acid C
Precision	1.40	1.70	1.48	1.36	1.42	1.36	1.34	1.54
Stability	0.55	0.20	0.11	0.52	0.42	0.12	0.33	0.97
Repeatability	2.72	1.99	1.15	2.61	2.21	2.28	2.61	2.70
Accuracy	98.31	98.58	99.73	96.32	101.44	99.67	101.79	99.44

Table S8. RCFs in different instruments and columns.

Instrument	Column	RCF					
		237 nm		327 nm			
		Phillyrin	Arctiin	Neochlorogenic acid	Chlorogenic acid	Isochlorogenic acid A	Isochlorogenic acid C
Thermo UltiMate3000-1	Hypersil ODS2	0.836	0.856	2.190	1.611	1.564	1.782
	Thermo Syncronis C18	0.832	0.865	2.187	1.611	1.566	1.795
Thermo UltiMate3000-2	Hypersil ODS2	0.838	0.867	2.187	1.614	1.566	1.795
	Thermo Syncronis C18	0.839	0.856	2.186	1.612	1.564	1.776
Mean		0.836	0.861	2.188	1.612	1.565	1.787
RSD		0.37%	0.68%	0.08%	0.09%	0.07%	0.53%
